# Guppies Prefer to Follow Large (Robot) Leaders Irrespective of Own Size

**DOI:** 10.3389/fbioe.2020.00441

**Published:** 2020-05-15

**Authors:** David Bierbach, Hauke J. Mönck, Juliane Lukas, Marie Habedank, Pawel Romanczuk, Tim Landgraf, Jens Krause

**Affiliations:** ^1^Faculty of Life Sciences, Thaer Institute, Humboldt-Universität zu Berlin, Berlin, Germany; ^2^Excellence Cluster ‘Science of Intelligence’, Technische Universität Berlin, Berlin, Germany; ^3^Department of Biology and Ecology of Fishes, Leibniz-Institute of Freshwater Ecology and Inland Fisheries, Berlin, Germany; ^4^Department of Mathematics and Computer Science, Institute for Computer Science, Freie Universität Berlin, Berlin, Germany; ^5^Department of Biology, Institute for Theoretical Biology, Humboldt-Universität zu Berlin, Berlin, Germany; ^6^Bernstein Center for Computational Neuroscience, Humboldt-Universität zu Berlin, Berlin, Germany

**Keywords:** biomimetic robots, *Poecilia reticulata*, leadership, body size, robotic fish

## Abstract

Body size is often assumed to determine how successfully an individual can lead others with larger individuals being better leaders than smaller ones. But even if larger individuals are more readily followed, body size often correlates with specific behavioral patterns and it is thus unclear whether larger individuals are more often followed than smaller ones because of their size or because they behave in a certain way. To control for behavioral differences among differentially-sized leaders, we used biomimetic robotic fish (Robofish) of different sizes. Live guppies (*Poecilia reticulata*) are known to interact with Robofish in a similar way as with live conspecifics. Consequently, Robofish may serve as a conspecific-like leader that provides standardized behaviors irrespective of its size. We asked whether larger Robofish leaders are preferentially followed and whether the preferences of followers depend on own body size or risk-taking behavior (“boldness”). We found that live female guppies followed larger Robofish leaders in closer proximity than smaller ones and this pattern was independent of the followers’ own body size as well as risk-taking behavior. Our study shows a “bigger is better” pattern in leadership that is independent of behavioral differences among differentially-sized leaders, followers’ own size and risk-taking behavior.

## Introduction

The question of what makes an individual successful in leading others is a long-standing issue (Krause et al., 2000; King et al., 2009). In shoaling fish, those individuals that occupy front or periphery positions within a shoal are assumed to have the greatest influence on the group’s movement direction, hence are capable of leading the other shoal members (Bumann and Krause, 1993; Krause et al., 2000; Lopez et al., 2012; Jolles et al., 2017). Often, occupation of front or peripheral positions is related to motivational or phenotypical differences among individuals (Krause et al., 2000; Hemelrijk and Kunz, 2005). For example, individuals that take up a front position are often hungrier (Krause et al., 1992; McLean et al., 2018), more risk-taking (“bolder”) (Leblond and Reebs, 2006; Nakayama et al., 2012a, b, 2016) or simply larger (Krause et al., 1998; Guttridge et al., 2011) than the rest of the group. Mechanistically, those front individuals may move faster (Gueron et al., 1996; Krause et al., 1998; Jolles et al., 2017) or have larger repulsion areas (Hemelrijk and Kunz, 2005; Romenskyy et al., 2017), both resulting in an assortment within the shoal. However, being at the front (i.e., taking the lead) is often not the only factor determining leadership success. Using the golden shiner (*Notemigonus crysoleucas*), Reebs (2001) showed that a minority of informed large fish was capable of leading a shoal of small fish to a food source, whereas informed small fish had much lower success in leading a shoal of large fish even when occupying the front positions of the shoal. Furthermore, when sticklebacks (*Gasterosteus aculeatus*) were grouped with two partners of different personalities, they were more likely to follow the partner of similar personality out of refuge (Nakayama et al., 2016). Thus, both body size as well as behavior may determine leadership success in fishes. Moreover, both body size and behavior often covary with each other, for example larger fish can swim faster (Domenici, 2001) than smaller ones or exhibit a certain personality (Polverino et al., 2016). Just recently Romenskyy et al. (2017) concluded that “fish of different sizes cannot be considered simply as particles of different physical size, since their behavior changes with their size.” This poses the question whether larger individuals are more often followed than smaller ones simply because they are larger or because they behave in a certain way. Furthermore, we do not know whether following behavior is influenced by the followers’ own body size or behavior, or how either attribute may interact with leader size. To answer these questions, we experimentally controlled for the leader’s behavior while simultaneously varying its body size through the use of differentially sized biomimetic robotic fish.

Biomimetic robots have become a recent tool to investigate animal behavior (Krause et al., 2011; Romano et al., 2018). These machines consist of an animal-like part that is either self-propelled or externally dragged by a robotic unit. Biomimetic robots can be either interactive (closed-loop behavior), which means that they change their behavior in response to the actions of live animals, or static (open-loop behavior), which means that they move and behave in predefined, non-interactive ways (Webb, 2000; Krause et al., 2011; Butail et al., 2015; Romano et al., 2018). Biomimetic robots thus provide the experimenter with a diverse toolset to study social interactions such as the ability to provide completely standardized social cues (e.g., through the use of non-interactive open-loop robots, see Abaid et al., 2012; Phamduy et al., 2014; Bierbach et al., 2018a). Furthermore, the robot’s parameters can be set to either resemble those of focal live individuals or show a sharp contrast with them (Butail et al., 2013, 2014; Polverino and Porfiri, 2013). In addition, closed-loop-controlled robots allow us to create interactive scenarios that nevertheless follow controlled rules that can be adapted intentionally (Kopman et al., 2013; Landgraf et al., 2013, 2014, 2016; Bonnet et al., 2018; Kim et al., 2018; Datteri, 2020).

To date a wide range of taxa has been shown to accept biomimetic robots as conspecific or heterospecific animals. For example, Halloy et al. (2007) developed a robot that interacted autonomously with live cockroaches and therefore allowed fine-scaled investigations of their aggregation behavior. Romano et al. (2017a) investigated the lateralization of escape and surveillance responses in locusts during predator–prey interactions with a robot that resembled a predatory bird. Similarly, fish shoals were attacked by a robotic predator fish to investigate their collective predator evasion responses (Swain et al., 2012; Romano et al., 2020). Such interactions can severely impact growth rates and body conditions of the attacked fish, even when experiencing these attacks only for short amounts of time (Polverino et al., 2019). These studies exemplify the extensive use of different fish species in studies with biomimetic robots (but see Romano et al. (2018)) for a more complete list of taxa). More or less natural interaction patterns among live fish and biomimetic robots have now been reported for poeciliids (Polverino and Porfiri, 2013; Bierbach et al., 2018a, b; Heathcote et al., 2018), killifish (Phamduy et al., 2014), zebrafish (Kim et al., 2018), golden shiners (Abaid et al., 2013), mormyrids (Donati et al., 2016; Worm et al., 2018), Siamese fighting fish (Romano et al., 2017b) as well as sticklebacks (Faria et al., 2010).

Their success in being accepted as conspecific or heterospecific animals may be due to Nico Tinbergen’s (1948) idea of “social releasers,” meaning that only a small subset of perceivable cues are communicative signals. Thus, even minimalistic robot models can exploit species-specific cues that identify conspecifics or heterospecifics (see Landgraf et al., 2016; Datteri, 2020 for discussion). In Halloy et al. (2007), for example, the robot was treated with a cockroach-specific pheromone to facilitate group integration. In poeciliids like the herein used guppies, replicas equipped with realistic glass eyes were found to be followed almost as close as live conspecific partners (Landgraf et al., 2016).

In the current study, we used the so-called Robofish system which is an open-loop controlled (e.g., non-interactive) robot platform that steers an exchangeable 3D-printed fish dummy. In our case, the replica resembled a live female Trinidadian guppy (*Poecilia reticulata*) (Landgraf et al., 2016). It was recently shown that live guppies interact similarly with Robofish as they do with a live conspecific (Bierbach et al., 2018b). Further, live guppies maintained individual differences in followership patterns exhibited during Robofish trials even when tested consecutively with a live conspecifics (Bierbach et al., 2018b).

We tested differentially-sized live female guppies for their risk-taking behavior (i.e., time to leave a shelter box) and their tendency to follow one of three differentially-sized, robotically steered replicas that all moved almost identically on a predefined trajectory in a large experimental tank. We asked (a) whether larger Robofish leaders are preferentially followed (assuming “bigger is better”), and (b) whether the following tendencies of followers depend on their own body size or their risk taking behavior (“boldness”). As in previous studies (Landgraf et al., 2016; Bierbach et al., 2018a, b), we assumed stronger following tendencies when live fish kept shorter average distances toward to moving Robofish.

## Methods

### Study Organism and Maintenance

We used female Trinidadian guppies (*Poecilia reticulata*) that were descendants of wild-caught fish from the Arima River in North Trinidad. Test fish came from large, randomly outbred single-species stocks maintained at the animal care facilities at the Faculty of Life Sciences, Humboldt University of Berlin. We provided a natural 12:12 h light:dark regime and maintained water temperature at 26°C. Fish were fed twice daily *ad libitum* with commercially available flake food (TetraMin^TM^) and once a week with frozen *Artemia* shrimps.

### The Robofish System

**FIGURE 1:**
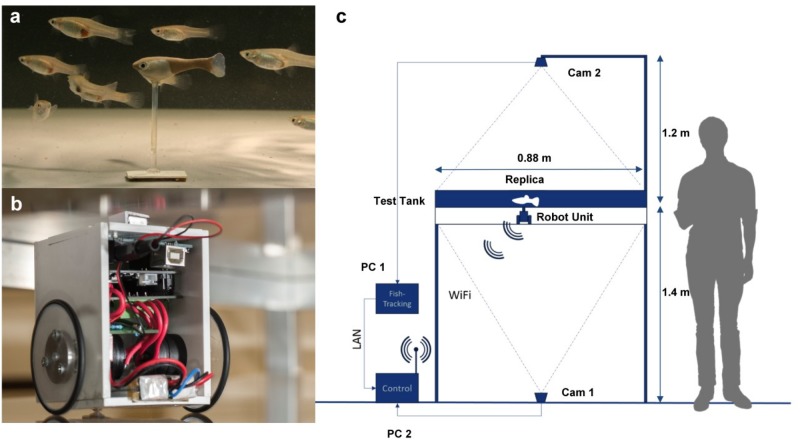
The Robofish system. **(a)** Guppy-like replica (3D printed and colored) with a group of female guppies in the test arena. **(b)** Close-up of the robot unit. The robot unit is driving on a second level below the test arena **(c)**.

The Robofish is a three-dimensional (3D)-printed guppy-like replica that is attached to a magnetic base. The magnetic base aligns with a wheeled robot that is driving below the actual test tank (88 × 88 cm, coated with white plastic foil) on a transparent second level. Hence, the replica can be moved directly by the robot (Figure 1). The entire system is enclosed in a black, opaque canvas to minimize exposure to external disturbances. The tank is illuminated from above with diffused LED lights. On the floor, a camera is facing upwards to track the robot’s movements through the transparent second level. A second camera is fixed above the tank to track both live fish and replicas. Two computers are used for system operation: one PC tracks the robot, computes and sends motion commands to the robot over a wireless channel. The second PC records the video feed of the second camera which is afterward tracked by custom-made software (Mönck et al., 2018). Please see our Supplementary Material for more details on the Robofish system as well as (Landgraf et al., 2016).

### Experimental Setup

To provide live female guppies with differently sized Robofish leaders, we used three replicas that differed only in body size [replica 1 = 20 mm standard length (SL); replica 2 = 25 mm SL, replica 3 = 30 mm SL, see Supplementary Figure S1]. All replicas were equipped with 3 mm glass eyes. Thus, the relative eye size decreased from the smallest to the largest replica (0.15–0.10). Such a decrease in relative eye size is common to all vertebrates including fishes (see Richardson et al., 2015) and thus our replicas mirrored the naturally found change in relative eye size during ontogeny. As we used transparent screws to attach the replica to its magnet foot, all replicas regardless of size kept the same distance to the water surface (1 cm, at 10 cm water level). We used only females to avoid sex differences in attraction toward Robofish, which resembles a female guppy. Test fish were randomly chosen to span the natural body size variation of this species (ranging from 18.0 to 32.0 mm, mean = 25.6 mm, *SD* = 4.1 mm, *n* = 88 fish used in this study). To measure body size, fish were transferred into a water-filled petri dish placed upon millimeter paper after the behavioral testing. We took a picture from centrally above using a SLR camera (Canon EOS 400D) and measured standard length of the fish from these pictures using ImageJ software (Schindelin et al., 2012). We tested 90 fish but had to remove 2 fish from the analysis as the video recording failed due to technical issues.

**FIGURE 2:**
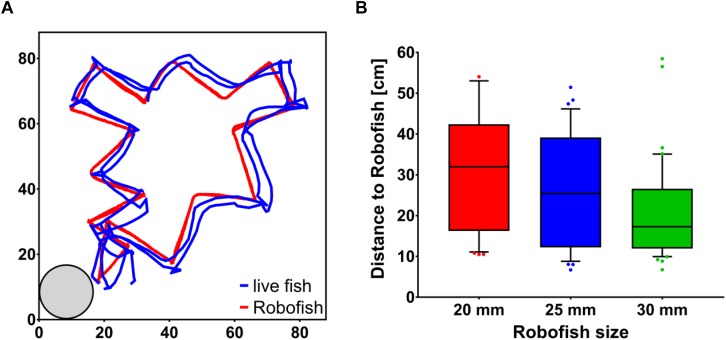
Following behavior of live guppies toward differently sized Robofish replicas. **(A)** Example track of a trial with Robofish. Fish were introduced into the start box (gray circle, lower left corner) and released into the tank after 1 min. Robofish then moved on a predefined zig-zag trajectory through the tank until it reached its start position. This movement was repeated a second time and a trial lasted about 60 s in total. **(B)** Inter-individual distance (Distance to Robofish) between live fish and differentially-sized Robofish replicas. Shown are medians and interquartile ranges (whiskers at 10 and 90% percentiles, with outliers as dots).

To initiate a trial, we transferred individual test fish into an opaque PVC cylinder located at the lower left corner of the test tank. The PVC cylinder had an opening (diameter 3 cm), which was closed with a sponge. We removed the sponge after 1 min of acclimation and noted the time each fish took to leave the cylinder as a proxy for its risk-taking tendency (i.e., “boldness”), which is thought to correlate with following tendencies (Nakayama et al., 2012a, b, 2016; Jolles et al., 2015). We initiated the Robofish’s movement sequence when the live fish left the cylinder (i.e., one body length away from the cylinder’s border). Robofish moved along a zig-zag pattern with a maximum speed of 15 cm/s and reduced its speed at the turning points to almost 0 cm/s, before accelerating again to the predefined maximum speed. This stop-and-go motion pattern led to an average speed of 10 cm/s. A zigzag movement was found to increase the likelihood of the Robofish to be followed (Landgraf et al., 2016) and the differently-sized replicas did not differ in exhibited velocities (see Supplementary Material). During the trials, Robofish moved to the opposite corner and then counter-clockwise to its start position. This round was repeated for a second time and a trial took about 60 s in total (see Figure 2 for an example track as well as Supplementary Video S1). Each trial was videotaped for subsequent tracking and the test fish was transferred back to its holding tank after size measurement was completed. Videos were recorded at 30 fps and also tracking was performed at the same sampling frame rate via Biotracker (Mönck et al., 2018). We analyzed the first 50 s after the fish left the shelter box, resulting in 1,500 frames analyzed per trial. We calculated the inter-individual distance between focal fish and Robofish as the average distance between subjects for all 1,500 frames. IID has been shown to reflect a live fish’s tendency to follow the moving Robofish (Landgraf et al., 2016; Bierbach et al., 2018a, b).

### Statistical Analysis

We initially log10 transformed both recorded continuous response variables (IID, time to leave start box) to match a Gaussian distribution. We then used the IID as dependent variable in an ANCOVA (unianova package in SPSS 25) with “leader size” as a fixed factor. We included “live fish body size (SL in mm)” and “time to leave start box [log10(s)]” as well as their interactions with “leader size” as covariates in the model. Non-significant interaction terms were removed from the final model. In order to test whether differently-sized live fish differ in their risk aversion tendency, we further correlated live fish body size (SL in mm) with time to leave shelter [log10 (s)] using Pearson’s correlation.

## Results

Regardless of own size [non-significant covariate “live fish body size” *F*_(1, 83)_ = 3.36; *p* = 0.071], live guppies tested with larger Robofish replicas followed significantly closer than those tested with smaller ones [significant effect of factor “leader size” *F*_(2, 78)_ = 4.49; *p* = 0.009, Figure 2]. There was no size assortative pattern detectable (i.e., smaller live fish did not follow smaller replicas closer than larger ones and *vice versa*) as suggested by a non-significant interaction term “leader size × live fish body size” [*F*_(4, 79)_ = 0.49; *p* = 0.62]. Also, the time each fish took to leave the start box had no significant influence on its following behavior [*F*_(1, 83)_ = 0.98; *p* = 0.33] and there was no significant interaction with leader size detectable [*F*_(2, 79)_ = 1.51; *p* = 0.23]. We found no significant correlation between live fish’s body size and their tendency to leave the start box (Pearsons’s *r* = 0.190, *p* = 0.073).

## Discussion

Live guppies followed larger Robofish leaders closer than smaller ones and this pattern was independent of the followers’ own body size as well as risk-taking behavior. This result is consistent with a study using golden shiners that found large individuals are more readily followed than small ones (Reebs, 2001). However, this is the first study that showed a “bigger is better” pattern in leadership in shoaling fish while controlling for the potentially confounding effects of behavioral differences (in small and large fish) by using a biomimetic robot.

Body size in fishes is often inevitably linked to specific behavioral patterns (Domenici, 2001; Polverino et al., 2016) and it is thus experimentally difficult to disentangle which cue (body size or linked behavior) is used by individuals that have to choose whom to follow among conspecifics of different sizes. While researchers from the field of sexual selection make use of video animations in binary choice tests to decouple behavior from body size and keep either one constant while varying the other (see Fisher and Rosenthal, 2007; Fisher et al., 2009; Gierszewski et al., 2017; Sommer-Trembo et al., 2017), the study of collective movement has largely relied on the use of live stimuli (but see Stowers et al., 2017 for a working Virtual Reality set-up). We addressed this issue by using a biomimetic robot toward live guppies show natural interaction patterns (Landgraf et al., 2016). Still, open-loop controlled robots (e.g., those that do not adjust their behavior in real-time to the actions of a partner) may have some short-comings in terms of reproducing natural behavioral feedbacks (see discussion in Bierbach et al., 2018b), they provide a powerful tool to present live animals with a standardized and almost identical set of social cues in a full contact design (i.e., not only visual cues available). It is thus possible to control for differences in leaders’ behavior that may affect leadership success like swimming speed (Jolles et al., 2017) and movement patterns (Ioannou et al., 2015; Nakayama et al., 2016), while simultaneously varying the parameter of interest (e.g., body size of the leader).

Our results show that live guppies followed larger Robofish closer than smaller ones and there are several (although not mutually exclusive) explanations for this result: (a) Larger individuals are often focused on by predators (Pocklington and Dill, 1995) and, in case of poeciliid females, experience more harassment by males (Herdman et al., 2004; Agrillo et al., 2006). Thus, small female guppies can benefit from associating with larger ones, as doing so may take away the attention of predators and/or harassing males. Larger individuals, however, can benefit from associating with other large individuals to minimize the oddity effect during predation (Peuhkuri, 1997; Krause and Ruxton, 2002). (b) Larger individuals in species with continuous growth throughout their lives are older and thus more experienced than smaller, younger individuals. As most teleost fishes show indeterminate growth, larger and thus older ones may have accumulated experience that provides them with fitness benefits, for example in terms of predator recognition (Brown and Smith, 1998; Holmes and McCormick, 2010) and navigation (Odling-Smee and Braithwaite, 2003). It might thus be beneficial for followers (regardless of own size) to associate with those experienced, larger phenotypes. This concept is also assumed to be important in the context of social learning (see Laland, 2004). For example, younger thus smaller guppy females copy the mate choice of older thus larger ones more readily (Dugatkin and Godin, 1993; Amlacher and Dugatkin, 2005). (c) Larger individuals are more conspicuous than smaller ones (both visual and non-visual, see Faria et al., 2010). As staying together as a group is assumed beneficial (Krause and Ruxton, 2002) and environmental conditions such as water turbidity (Borner et al., 2015) often hamper prober sensing of conspecifics, following the larger, more conspicuous ones might be under positive selection.

Live fish’s own body size did not affect following tendencies in our study, and although we found no evidence for size-assortative following, sorting by size is common in fish shoals in the wild (Hoare et al., 2000). While fish might have a ubiquitous preference to follow larger individuals, assortative patterns might simply arise mechanistically because those front-occupying individuals may swim faster (Krause et al., 2000) leading to an assortment within the shoal (Hoare et al., 2000). Also other factors may contribute to observed size assortative patterns, most likely mediated through competition among group members (Hoare et al., 2000; Croft et al., 2009). Body size in many species correlates with an individual’s fighting ability and, as a result, its dominance status (Arnott and Elwood, 2009). In turn, smaller, inferior individuals despite their preference to associate with larger, dominant conspecifics, may suffer from increased aggressive behaviors, which, ultimately, can lead to exclusion from the group (Magurran and Seghers, 1991; Hoare et al., 2000). However, in Atlantic mollies (*Poecilia mexicana*), a species closely related to the guppy, size-assortative patterns in both sexes have been found to arise only gradually over time, e.g., a size-assortative pattern was only detectable after a few days of familiarization (Bierbach et al., 2014). Thus, despite a possible preference to associate and/or follower larger individuals, there are many other factors that can lead to observable size-assortative patterns in the wild (Hoare et al., 2000; Croft et al., 2009).

We found no evidence that follower’s risk-taking behavior affected their tendencies to follow Robofish leaders of different size. This result is in contrast to studies in sticklebacks, where shyer individuals are better followers and are less likely to initiate leadership behavior themselves (Leblond and Reebs, 2006; Nakayama et al., 2012a, b, 2016). Besides possible species-specific differences, reinforcing feedbacks due to mutual influences among leaders and followers may have led to the observed personality-dependent following behavior in sticklebacks (Harcourt et al., 2009). Future comparative studies will thus help to pinpoint species-specific leadership behaviors as well as general patterns observable across taxa. However, this would need experimenters to take the same experimental approach while investigating a variety of different species – at least for small fish a platform like Robofish or similar tools that allow experimenters to adapt their systems quickly to different species (i.e., by exchanging the replicas) seem to be a promising tool for this.

A leader’s behavior is clearly influencing its leadership success (Ioannou et al., 2015; Nakayama et al., 2016), but independent of size-specific behavioral differences, body size seems to be the sole determinant of success in leading others in a “bigger is better” way at least in the guppy. When followers have ubiquitous preferences for large-bodied leaders, theoretical and practical considerations of collective behavior will strongly benefit from accounting for these size-specific leadership patterns.

Biomimetic robots allow experimenters to gain control of the animal stimulus, which is not possible using live animals. This may have several potential benefits for the study of animal behavior: First, standardized test assays become available that minimize variation of provided stimuli which might come into play when using live animals as social stimulus. Second, decoupling of behavior and morphology becomes available as we demonstrated in the current study. Third, interactive robots can validate theoretically proposed mechanisms of social interactions among animals as their rules of interaction can be systematically manipulated. Forth, the use of artificial stimuli can reduce the number of individuals tested during experimentation (though same or better data quality is achieved through highly standardized test procedures) and thus help to promote the 3R principle in behavioral research. In fact, future developments may further increase the usage of biomimetic robots for example through use of soft robotic techniques (Li et al., 2017; Katzschmann et al., 2018; Gao et al., 2019) or a better integration of acoustic or olfactory cues which are important communication channels in many species, including fish (Ward et al., 2002; Bass and McKibben, 2003; Halloy et al., 2007). Biomimetic robots, together with video animations or virtual reality platforms, are thus able to strongly assist biologists answering a wide variety of question that could not be answered through the use of classical experimental assays.

## Data Availability Statement

All datasets generated for this study are included in the article/Supplementary Material.

## Ethics Statement

The experiments reported here comply with the current German laws approved by LaGeSo Berlin (Reg. 0117/16 to DB).

## Author Contributions

DB and JK designed the study. DB, HM, JK, and TL built the Robofish system. DB, HM, MH, and JL performed the experiments. DB and PR analyzed the data. DB wrote the first draft of the manuscript. All authors contributed to the final version of the manuscript and approved the submission.

## Conflict of Interest

The authors declare that the research was conducted in the absence of any commercial or financial relationships that could be construed as a potential conflict of interest.

## Supplementary Material

FIGURE S1 Photograph of differently-sized replicas. Left (20 mm SL) and middle (25 mm SL) replicas are unprocessed 3D printed blanks that were later on equipped with glass eyes and color-painted as shown for the 30 mm replica on the right.

FIGURE S2 Speed distributions during a trial **(a)** as well as speed profiles **(b)** of the three differently-sized replicas. In **(a)** fraction of speeds with a 1 cm/s bin size are shown for a trial run (60 s). There was no significant difference among replicas (Kolmogorov-Smirnoff test: *P* > 0.41). In **(b)** the speed profiles of Robofish with all three replicas are shown. There was no significant difference in accelerating/decelerating among replicas (Kolmogorov-Smirnoff test: *P* > 0.99). Please note that this pattern of acceleration and deceleration is typically found between two turning points along the zig-zag movement. Maximum speed was set to 15 cm/s (dashed line).

VIDEO S1 Example track of a live guppy following the Robofish replica.

## References

[B1] Abaid N. Bartolini T. Macri S. Porfiri M. (2012). Zebrafish responds differentially to a robotic fish of varying aspect ratio, tail beat frequency, noise, and color. *Behav. Brain Res.* 233 545–553. 10.1016/j.bbr.2012.05.047 22677270

[B2] Abaid N. Marras S. Fitzgibbons C. Porfiri M. (2013). Modulation of risk-taking behaviour in golden shiners (*Notemigonus crysoleucas*) using robotic fish. *Behav. Processes* 100 9–12. 10.1016/j.beproc.2013.07.010 23876393

[B3] Agrillo C. Dadda M. Bisazza A. (2006). Sexual harassment influences group choice in female mosquitofish. *Ethology* 112 592–598. 10.1111/j.1439-0310.2006.01188.x

[B4] Amlacher J. Dugatkin L. A. (2005). Preference for older over younger models during mate-choice copying in young guppies. *Ethol. Ecol. Evol.* 17 161–169. 10.1080/08927014.2005.9522605

[B5] Arnott G. Elwood R. W. (2009). Assessment of fighting ability in animal contests. *Anim. Behav.* 77 991–1004.

[B6] Bass A. H. McKibben J. R. (2003). Neural mechanisms and behaviors for acoustic communication in teleost fish. *Prog. Neurobiol.* 69 1–26. 10.1016/S0301-0082(03)00004-2) 12637170

[B7] Bierbach D. Lukas J. Bergmann A. Elsner K. Höhne L. Weber C. (2018a). Insights into the social behavior of surface and cave-dwelling fish (*Poecilia mexicana*) in light and darkness through the use of a biomimetic robot. *Front. Robot. AI* 5:3 10.3389/frobt.2018.00003 PMC7805783 33500890

[B8] Bierbach D. Landgraf T. Romanczuk P. Lukas J. Nguyen H. Wolf M. (2018b). Using a robotic fish to investigate individual differences in social responsiveness in the guppy. *R. Soc. Open Sci.* 5:181026. 10.1098/rsos.181026 30225087 PMC6124066

[B9] Bierbach D. Mönck H. J. Lukas J. Habedank M. Romanczuk P. Landgraf T. (2018c). Guppies prefer to follow large (robot) leaders irrespective of own size. *bioRxiv* [Preprint]. 10.1101/320911 PMC7243707 32500065

[B10] Bierbach D. Oster S. Jourdan J. Arias-Rodriguez L. Krause J. Wilson A. M. (2014). Social network analysis resolves temporal dynamics of male dominance relationships. *Behav. Ecol. Sociobiol.* 68 935–945.

[B11] Bonnet F. Gribovskiy A. Halloy J. Mondada F. (2018). Closed-loop interactions between a shoal of zebrafish and a group of robotic fish in a circular corridor. *Swarm Intell.* 12 227–244. 10.1007/s11721-017-0153-6

[B12] Borner K. K. Krause S. Mehner T. Uusi-Heikkilä S. Ramnarine I. W. Krause J. (2015). Turbidity affects social dynamics in Trinidadian guppies. *Behav. Ecol. Sociobiol.* 69 645–651.

[B13] Brown G. E. Smith R. J. F. (1998). Acquired predator recognition in juvenile rainbow trout (*Oncorhynchus mykiss*): conditioning hatchery-reared fish to recognize chemical cues of a predator. *Can. J. Fish. Aquatc. Sci.* 55 611–617.

[B14] Bumann D. Krause J. (1993). Front individuals lead in shoals of three-spined sticklebacks (*Gasterosteus aculeatus*) and juvenile roach (*Rutilus rutilus*). *Behaviour* 125 189–198. 10.1163/156853993X00236

[B15] Butail S. Abaid N. Macri S. Porfiri M. (2015). “Fish–robot interactions: robot fish in animal behavioral studies,” in *Robot Fish*, eds Du R. Li Z. Youcef-Toumi K. Valdivia P. Alvarado (Berlin: Springer), 359–377.

[B16] Butail S. Bartolini T. Porfiri M. (2013). Collective response of zebrafish shoals to a free-swimming robotic fish. *PLoS One* 8:e76123 10.1371/journal.pone.0076123 PMC3797741 24146825

[B17] Butail S. Polverino G. Phamduy P. Del Sette F. Porfiri M. (2014). Influence of robotic shoal size, configuration, and activity on zebrafish behavior in a free-swimming environment. *Behav. Brain Res.* 275 269–280. 10.1016/j.bbr.2014.09.015 25239605

[B18] Croft D. P. Darden S. K. Ruxton G. D. (2009). Predation risk as a driving force for phenotypic assortment: a cross-population comparison. *Proc. R. Soc. B Biol. Sci.* 276 1899–1904. 10.1098/rspb.2008.1928 19324770 PMC2674500

[B19] Datteri E. (2020). Interactive biorobotics. *Synthese* 10.1007/s11229-020-02533-2 PMC7360728 32733858

[B20] Domenici P. (2001). The scaling of locomotor performance in predator–prey encounters: from fish to killer whales. *Comp. Biochem. Physiol. Part A Mol. Integr. Physiol.* 131 169–182. 10.1016/S1095-6433(01)00465-2 11733175

[B21] Donati E. Worm M. Mintchev S. Wiel M. V. D. Benelli G. Emde G. V. D. (2016). Investigation of collective behaviour and electrocommunication in the weakly electric fish, *Mormyrus rume*, through a biomimetic robotic dummy Fish. *Bioinspir. Biomim.* 11:066009 10.1088/1748-3190/11/6/066009 27906686

[B22] Dugatkin L. A. Godin J.-G. J. (1993). Female mate copying in the guppy (*Poecilia reticulata*): age-dependent effects. *Behav. Ecol.* 4 289–292. 10.1093/beheco/4.4.289

[B23] Faria J. J. Dyer J. R. G. Clément R. O. Couzin I. D. Holt N. Ward A. J. W. (2010). A novel method for investigating the collective behaviour of fish: Introducing ‘Robofish’. *Behav. Ecol. Sociobiol.* 64 1211–1218.

[B24] Fisher H. S. Mascuch S. J. Rosenthal G. G. (2009). Multivariate male traits misalign with multivariate female preferences in the swordtail fish, *Xiphophorus birchmanni*. *Anim. Behav.* 78 265–269. 10.1016/j.anbehav.2009.02.029

[B25] Fisher H. S. Rosenthal G. G. (2007). Male swordtails court with an audience in mind. *Biol. Lett.* 3 5–7. 10.1098/rsbl.2006.0556 17443951 PMC2373807

[B26] Gao Z. Shi Q. Fukuda T. Li C. Huang Q. (2019). An overview of biomimetic robots with animal behaviors. *Neurocomputing* 332 339–350. 10.1016/j.neucom.2018.12.071

[B27] Gierszewski S. Müller K. Smielik I. Hütwohl J.-M. Kuhnert K.-D. Witte K. (2017). The virtual lover: variable and easily guided 3D fish animations as an innovative tool in mate-choice experiments with sailfin mollies-II, Validation. *Curr. Zool.* 63 65–74. 10.1093/cz/zow108 29491964 PMC5804156

[B28] Gueron S. Levin S. A. Rubenstein D. I. (1996). The dynamics of herds: from individuals to aggregations. *J. Theor. Biol.* 182 85–98. 10.1006/jtbi.1996.0144 24312727

[B29] Guttridge T. L. Gruber S. H. DiBattista J. D. Feldheim K. A. Croft D. P. Krause S. (2011). Assortative interactions and leadership in a free-ranging population of juvenile lemon shark *Negaprion brevirostris*. *Mar. Ecol. Prog. Ser.* 423 235–245.

[B30] Halloy J. Sempo G. Caprari G. Rivault C. Asadpour M. Tache F. (2007). Social integration of robots into groups of cockroaches to control self-organized choices. *Science* 318 1155–1158. 10.1126/science.1144259 18006751

[B31] Harcourt J. L. Ang T. Z. Sweetman G. Johnstone R. A. Manica A. (2009). Social feedback and the eEmergence of leaders and followers. *Curr. Biol.* 19 248–252. 10.1016/j.cub.2008.12.051 19185497

[B32] Heathcote R. J. P. Darden S. K. Troscianko J. Lawson M. R. M. Brown A. M. Laker P. R. (2018). Dynamic eye colour as an honest signal of aggression. *Curr. Biol.* 28 R652–R653. 10.1016/j.cub.2018.04.078 29870700

[B33] Hemelrijk C. K. Kunz H. (2005). Density distribution and size sorting in fish schools: an individual-based model. *Behav. Ecol.* 16 178–187. 10.1093/beheco/arh149

[B34] Herdman E. J. E. Kelly C. D. Godin J.-G. J. (2004). Male mate choice in the guppy (*Poecilia reticulata*): Do males prefer larger females as mates? *Ethology* 110 97–111. 10.1111/j.1439-0310.2003.00960.x

[B35] Hoare D. J. Krause J. Peuhkuri N. Godin J. G. J. (2000). Body size and shoaling in fish. *J. Fish Biol.* 57 1351–1366. 10.1111/j.1095-8649.2000.tb02217.x

[B36] Holmes T. H. McCormick M. I. (2010). Smell, learn and live: the role of chemical alarm cues in predator learning during early life history in a marine fish. *Behav. Process* 83 299–305. 10.1016/j.beproc.2010.01.013 20117187

[B37] Ioannou C. C. Singh M. Couzin I. D. (2015). Potential leaders trade off goal-oriented and socially oriented behavior in mobile animal groups. *Am. Nat.* 186 284–293. 10.1086/681988 26655156

[B38] Jolles J. W. Boogert N. J. Sridhar V. H. Couzin I. D. Manica A. (2017). Consistent individual differences drive collective behavior and group functioning of schooling fish. *Curr. Biol.* 27 2862–2868.e7. 10.1016/j.cub.2017.08.004 28889975 PMC5628957

[B39] Jolles J. W. Fleetwood-Wilson A. Nakayama S. Stumpe M. C. Johnstone R. A. Manica A. (2015). The role of social attraction and its link with boldness in the collective movements of three-spined sticklebacks. *Anim. Behav.* 99 147–153. 10.1016/j.anbehav.2014.11.004 25598543 PMC4289919

[B40] Katzschmann R. K. DelPreto J. MacCurdy R. Rus D. (2018). Exploration of underwater life with an acoustically controlled soft robotic fish. *Sci. Robot.* 3:eaar3449 10.1126/scirobotics.aar3449 33141748

[B41] Kim C. Ruberto T. Phamduy P. Porfiri M. (2018). Closed-loop control of zebrafish behaviour in three dimensions using a robotic stimulus. *Sci. Rep.* 8:657. 10.1038/s41598-017-19083-2 29330523 PMC5766612

[B42] King A. J. Johnson D. D. P. Van Vugt M. (2009). The origins and evolution of leadership. *Curr. Biol.* 19 R911–R916. 10.1016/j.cub.2009.07.027 19825357

[B43] Kopman V. Laut J. Polverino G. Porfiri M. (2013). Closed-loop control of zebrafish response using a bioinspired robotic-fish in a preference test. *J. R. Soc. Interface* 10:20120540 10.1098/rsif.2012.0540 PMC3565779 23152102

[B44] Krause J. Bumann D. Todt D. (1992). Relationship between the position preference and nutritional state of individuals in schools of juvenile roach (*Rutilus rutilus*). *Behav. Ecol. Sociobiol.* 30 177–180. 10.1007/bf00166700

[B45] Krause J. Hoare D. Krause S. Hemelrijk C. K. Rubenstein D. I. (2000). Leadership in fish shoals. *Fish Fish.* 1 82–89. 10.1111/j.1467-2979.2000.tb00001.x

[B46] Krause J. Reeves P. Hoare D. (1998). Positioning behaviour in roach shoals: the role of body length and nutritional state. *Behaviour* 135 1031–1039. 10.1163/156853998792913519

[B47] Krause J. Ruxton G. D. (2002). *Living in Groups.* Oxford: Oxford University Press.

[B48] Krause J. Winfield A. F. T. Deneubourg J.-L. (2011). Interactive robots in experimental biology. *Trends Ecol. Evol.* 26 369–375. 10.1016/j.tree.2011.03.015 21496942

[B49] Laland K. (2004). Social learning strategies. *Anim. Learn. Behav.* 32 4–14. 10.3758/BF03196002 15161136

[B50] Landgraf T. Bierbach D. Nguyen H. Muggelberg N. Romanczuk P. Krause J. (2016). RoboFish: increased acceptance of interactive robotic fish with realistic eyes and natural motion patterns by live Trinidadian guppies. *Bioinspir. Biomim.* 11:015001. 10.1088/1748-3190/11/1/015001 26757096

[B51] Landgraf T. Nguyen H. Forgo S. Schneider J. Schröer J. Krüger C. (2013). “Interactive robotic fish for the analysis of swarm behavior,” in *Advances in Swarm Intelligence*, eds Tan Y. Shi Y. Mo H. (Heidelberg: Springer), 1–10.

[B52] Landgraf T. Nguyen H. Schröer J. Szengel A. Clément R. G. Bierbach D. (2014). “Blending in with the shoal: robotic fish swarms for investigating strategies of group formation in guppies,” in *Biomimetic and Biohybrid Systems*, eds Duff A. Lepora N. Mura A. Prescott T. Verschure P. M. J. (Basel: Springer International Publishing), 178–189.

[B53] Leblond C. Reebs S. G. (2006). Individual leadership and boldness in shoals of golden shiners. *Behaviour* 143 1263–1280. 10.1163/156853906778691603

[B54] Li T. Li G. Liang Y. Cheng T. Dai J. Yang X. (2017). Fast-moving soft electronic fish. *Sci. Adv.* 3:e1602045. 10.1126/sciadv.1602045 28435879 PMC5381956

[B55] Lopez U. Gautrais J. Couzin I. D. Theraulaz G. (2012). From behavioural analyses to models of collective motion in fish schools. *Interface Focus* 2 693–707. 10.1098/rsfs.2012.0033 24312723 PMC3499128

[B56] Magurran A. E. Seghers B. H. (1991). Variation in schooling and aggression amongst guppy (*Poecilia reticulata*) populations in Trinidad. *Behaviour* 118 214–234.

[B57] McLean S. Persson A. Norin T. Killen S. S. (2018). Metabolic costs of feeding predictively alter the spatial distribution of individuals in fish schools. *Curr. Biol.* 28 1144–1149.e4. 10.1016/j.cub.2018.02.043 29576472

[B58] Mönck H. J. Jörg A. Falkenhausen T. V. Tanke J. Wild B. Dormagen D. (2018). BioTracker: an open-source computer vision framework for visual animal tracking. *arXiv* [Preprint]. Available online at: https://arxiv.org/abs/1803.07985 (accessed April 29, 2020).

[B59] Nakayama S. Harcourt J. L. Johnstone R. A. Manica A. (2012a). Initiative, personality and leadership in pairs of foraging fish. *PLoS One* 7:e36606. 10.1371/journal.pone.0036606 22567168 PMC3342251

[B60] Nakayama S. Johnstone R. A. Manica A. (2012b). Temperament and hunger interact to determine the emergence of leaders in pairs of foraging fish. *PLoS One* 7:e43747. 10.1371/journal.pone.0043747 22952753 PMC3430686

[B61] Nakayama S. Harcourt J. L. Johnstone R. A. Manica A. (2016). Who directs group movement? Leader effort versus follower preference in stickleback fish of different personality. *Biol. Lett.* 12:20160207 10.1098/rsbl.2016.0207 PMC4892248 27194292

[B62] Odling-Smee L. Braithwaite V. A. (2003). The role of learning in fish orientation. *Fish Fish.* 4 235–246. 10.1098/rsbl.2015.0937 26763221 PMC4785932

[B63] Peuhkuri N. (1997). Size-assortative shoaling in fish: the effect of oddity on foraging behaviour. *Anim. Behav.* 54 271–278. 10.1006/anbe.1996.0453 9268457

[B64] Phamduy P. Polverino G. Fuller R. C. Porfiri M. (2014). Fish and robot dancing together: bluefin killifish females respond differently to the courtship of a robot with varying color morphs. *Bioinspir. Biomim.* 9:036021 10.1088/1748-3182/9/3/036021 25162832

[B65] Pocklington R. Dill L. (1995). Predation on females or males: who pays for bright male traits? *Anim. Behav.* 49 1122–1124.

[B66] Polverino G. Bierbach D. Killen S. S. Uusi-Heikkilä S. Arlinghaus R. (2016). Body length rather than routine metabolic rate and body condition correlates with activity and risk-taking in juvenile zebrafish *Danio rerio*. *J. Fish Biol.* 89 2251–2267. 10.1111/jfb.13100 27615803 PMC6849769

[B67] Polverino G. Karakaya M. Spinello C. Soman V. R. Porfiri M. (2019). Behavioural and life-history responses of mosquitofish to biologically inspired and interactive robotic predators. *J. R. Soc. Interface* 16:20190359 10.1098/rsif.2019.0359 PMC6769303 31506048

[B68] Polverino G. Porfiri M. (2013). Mosquitofish (*Gambusia affinis*) responds differentially to a robotic fish of varying swimming depth and aspect ratio. *Behav. Brain Res.* 250 133–138. 10.1016/j.bbr.2013.05.008 23684918

[B69] Reebs S. G. (2001). Influence of body size on leadership in shoals of golden shiners, *Notemigonus crysoleucas*. *Behaviour* 138 797–809.

[B70] Richardson J. R. Shears N. T. Taylor R. B. (2015). Using relative eye size to estimate the length of fish from a single camera image. *Mar. Ecol. Prog. Ser.* 538 213–219. 10.3354/meps11476

[B71] Romano D. Benelli G. Stefanini C. (2017a). Escape and surveillance asymmetries in locusts exposed to a Guinea fowl-mimicking robot predator. *Sci. Rep.* 7:12825. 10.1038/s41598-017-12941-z 28993651 PMC5634469

[B72] Romano D. Benelli G. Donati E. Remorini D. Canale A. Stefanini C. (2017b). Multiple cues produced by a robotic fish modulate aggressive behaviour in Siamese fighting fishes. *Sci. Rep.* 7:4667 10.1038/s41598-017-04840-0 PMC5498610 28680126

[B73] Romano D. Donati E. Benelli G. Stefanini C. (2018). A review on animal–robot interaction: from bio-hybrid organisms to mixed societies. *Biol. Cybernet.* 113 201–225. 10.1007/s00422-018-0787-5 30430234

[B74] Romano D. Elayan H. Benelli G. Stefanini C. (2020). Together We Stand – Analyzing Schooling Behavior in Naive Newborn Guppies through Biorobotic Predators. *J. Bionic Eng.* 17 174–184. 10.1007/s42235-020-0014-7

[B75] Romenskyy M. Herbert-Read J. E. Ward A. J. W. Sumpter D. J. T. (2017). Body size affects the strength of social interactions and spatial organization of a schooling fish (*Pseudomugil signifer*). *R. Soc. Open Sci.* 4:161056. 10.1098/rsos.161056 28484622 PMC5414259

[B76] Schindelin J. Arganda-Carreras I. Frise E. (2012). Fiji: an open-source platform for biological-image analysis. *Nat. Methods* 9 676–682. 10.1038/nmeth.2019 22743772 PMC3855844

[B77] Sommer-Trembo C. Plath M. Gismann J. Helfrich C. Bierbach D. (2017). Context-dependent female mate choice maintains variation in male sexual activity. *R. Soc. Open Sci.* 4:170303. 10.1098/rsos.170303 28791157 PMC5541552

[B78] Stowers J. R. Hofbauer M. Bastien R. Griessner J. Higgins P. Farooqui S. (2017). Virtual reality for freely moving animals. *Nat. Methods* 14 995–1002. 10.1038/nmeth.4399 28825703 PMC6485657

[B79] Swain D. T. Couzin I. D. Leonard N. E. (2012). Real-time feedback-controlled robotic ?sh for behavioral experiments with ?sh schools. *Proc. IEEE* 100 150–163.

[B80] Ward A. J. Axford S. Krause J. (2002). Mixed-species shoaling in fish: the sensory mechanisms and costs of shoal choice. *Behav. Ecol. Sociobiol.* 52 182–187. 10.1007/s00265-002-0505-z

[B81] Webb B. (2000). What does robotics offer animal behaviour? *Anim. Behav.* 60 545–558. 10.1006/anbe.2000.1514 11082225

[B82] Worm M. Landgraf T. Prume J. Nguyen H. Kirschbaum F. von (2018). Evidence for mutual allocation of social attention through interactive signaling in a mormyrid weakly electric fish. *Proc. Natl. Acad. Sci. U.S.A.* 115 6852–6857. 10.1073/pnas.1801283115 29891707 PMC6042124

