# Supplementary material for: Guppies Prefer to Follow Large (Robot) Leaders Irrespective of Own Size
Source: Front Bioeng Biotechnol. 2020 May 15;8:441. doi: 10.3389/fbioe.2020.00441 (PMC7243707; doi:10.3389/fbioe.2020.00441)
Supplement: FIGURE S1 — Photograph of differently-sized replicas. Left (20 mm SL) and middle (25 mm SL) replicas are unprocessed 3D printed blanks that were later on equipped with glass eyes and color-painted as shown for the 30 mm replica on the right. [file Table_1.DOCX]

**Electronic Supplemental Material**

Guppies prefer to follow large (robot) leaders irrespective of own size

David Bierbach^*^, Hauke J. Mönck, Juliane Lukas, Marie Habedank, Pawel Romanczuk, Tim Landgraf, Jens Krause

*corresponding author: david.bierbach@gmx.de

**General description of the Robofish system**

The Robofish system consists of a squared glass tank (88×88 cm) that is filled to a level of 10 cm with aged tap water. The tank is placed on an aluminum rack at about 1.40m above ground. Our system allows simultaneous operation of multiple robots [1], however, for the current experiments only one robot was used at a time. The two-wheeled differential drive robots move below the tank on a transparent platform. Each robot carries a neodymium magnet directed to the bottom side of the tank. A 3D-printed fish replica is attached to a magnetic base, which aligns with the robot. Hence the replica can be moved directly by the robot. On the ground, a camera is facing upwards to track the robots. A second camera is fixed above the tank to track both live fish and replicas. The entire system is enclosed in a black, opaque canvas to minimize exposure to external disturbances. The tank is illuminated from above with artificial light reproducing the daylight spectrum.

Two personal computers are used for system operation: one PC tracks the robots, receives swarm tracking results from a second PC and computes and sends motion commands to each individual robot over a wireless channel. The second PC evaluates the video feed of the shoal camera, finds and tracks all relevant objects and sends those data to the first computer via a local area network connection [2].

**Robot design**

The two-wheeled robot is a cuboid with dimensions 7×7×10 cm built from aluminum and plastic plates. It houses one Arduino-compatible board for main processing and WiFi communication (ESP-12F). Custom-built wheels (diameter = 6 cm) with rubber gasket rings are directly connected to two dc gear motors (2622S006B SC 33:1, Dr Fritz Faulhaber GmbH & Co. KG) which include a speed controller for pulse width modulation (PWM) control. These controllers allow for precise speed actuation. The main power is supplied by a LiPo battery pack (two-cell, 7.4 V nominal output, Conrad Electronic SE). The motors are driven via PWM signals generated by the ESP-12F. A voltage divider scales down the raw battery output, which then can be measured on an analog pin of the ESP board with respect to an internal reference. The robot carries a neodymium magnet at the tip of a plastic rod held up against the glass tank maintaining an air gap. The tank itself is made of glass, which due to the mass of the water column bends slightly downwards. The distance between the magnet and the tank therefore varies from less than 1 mm near the center of the tank to approximately 2 mm in the periphery. The magnets’ poles are aligned in parallel to the motion plane and the replica’s base aligns with the robot. This steady coupling allows on-the-spot rotations and fast accelerations of the replica.

**Robot control and motion models**

The control software sends motion commands at a frequency of 25 Hz, as determined by the bottom camera’s frame rate. In each time step, a command packet is issued and sent to the individual robot via WiFi (UDP). Each robot has a unique IP-address and only receives its respective packets. We use a fixed length protocol with a 2 bytes header and 12 bytes data.

The motion control is organized in layers:

(1) Arbitrary behavior layer that generates global target points. These behaviors are required to implement a simple interface and are loaded as shared objects.

(2) Trajectory manipulation layer, which converts high level control commands into a command queue. Here motion types and navigation automation are done.

(3) The ego-motions layer, which converts the next command from the queue into motor speeds using a p/d/i controller with overlay speed-controlling. When operating on a command sequence the ego-motions layer (3) will check for every camera frame whether the current low-level target point has been reached (with a tolerance of 3 cm). If so, it pops this command from the queue.

The motors are able to propel the system to a maximum forward and turning velocity of approximately 60 cm/s and 860 deg/s, respectively. The minimum velocities are approximately 1 cm−s and 15 deg−s. No odometric feedback control has been used. The robots’ motion is solely controlled via vision feedback as to avoid drift. Most of the computation is outsourced to personal computers. Currently, the robot’s firmware implements the following: (1) Sending status packets back to the control computer at 1 Hz, including the robot’s unique identifier and battery level. (2) It generates PWM signals according to the received motor speed values. (3) It can be configured to run in jump-start mode. To overcome initial friction and inertia, this mode prepends higher PWM duty cycles when motor speeds are low.

**Fish Tracking**

The tracking algorithm and software presented in [2] have been ported into a plugin for the BioTracker [3] framework with minor changes to the algorithm itself. Interactions of robots and live fish are observed from above the tank by a second camera. In order to detect all individuals, we use a background subtraction procedure that models foreground and background pixel distributions as a mixture of Gaussians [4]. Since the tank’s bottom and walls are laminated with white plastic, the fish appear as clear dark objects in the video images. Once converged, the background model shows an empty tank such that the difference image of background and current live frame exhibits distinct positive peaks. The individual fish are detected by first applying a global threshold to the difference image. All regions having above-threshold values are then treated similarly to the blobs in the robot tracking. Additionally, the blobs are assigned an orientation by integrating the motion vector over a fixed time window, when fast movements are detected. The system assigns an ID to every fish blob and tracks it using an ellipsoid model.

**Replica construction and performance comparison**

The fish replicas were 3D-printed standard models (see [5, 6] for a similar approach). The 3D mesh was produced manually from several template photographs. The printed replicas were then painted and finished and equipped with glass eyes (3mm diameter, model: kristall B11; Lauschaer Glasaugen, Frank Weigelt). Replicas resemble female guppies as previous research showed that females were similarly preferred as shoaling partners by both sexes while males are often avoided by females due to the typically high rates of male sexual harassment (see [7]). We used three differentially-sized replicas: r1=20 mm standard length (SL); r2=25 mm SL, r3=30 mm SL (Figure S1). All measures represent standard length measured from snout to end of caudal peduncle.

For the current experiment, Robofish moved along a zig-zag pattern with a maximum speed of 15 cm/s (Video S1). The robot reduced its speed at the turning points to almost 0 cm/s while afterwards accelerating again to the predefined maximum speed. This stop-and-go motion pattern leads to an average speed of 10 cm/s. In order to ensure that differentially-sized replicas were moved almost identical by the robotic base, we compared speed distributions during a trial as well as speed profiles during acceleration/deceleration phases (between two turning points along the zig-zag movement). There was no significant difference among replicas in respect to speed distributions during a trial (Figure S2a, Kolmogorov-Smirnov tests: all P-values > 0.41) as well as in speed profiles (Figure S2b, K-S tests: all P-values > 0.99). These values were obtained from visual tracking of the Robofish movement thus represent the behavior that is visible to the live test fish as well.

**References**

1. Landgraf T., Nguyen H., Schröer J., Szengel A., Clément R.G., Bierbach D., Krause J. 2014 Blending in with the Shoal: Robotic Fish Swarms for Investigating Strategies of Group Formation in Guppies. In *Biomimetic and Biohybrid Systems* (eds. Duff A., Lepora N., Mura A., Prescott T., Verschure P.M.J.), pp. 178-189, Springer International Publishing.

2. Landgraf T., Bierbach D., Nguyen H., Muggelberg N., Romanczuk P., Krause J. 2016 RoboFish: increased acceptance of interactive robotic fish with realistic eyes and natural motion patterns by live Trinidadian guppies. *Bioinspir Biomim* **11**(1), 015001.

3. Mönck H.J., Jörg A., Falkenhausen T.v., Tanke J., Wild B., Dormagen D., Piotrowski J., Winklmayr C., Bierbach D., Landgraf T. 2018 BioTracker: An Open-Source Computer Vision Framework for Visual Animal Tracking. *arXiv:180307985*.

4. Zivkovic Z., Heijden F.v.d. 2004 Recursive unsupervised learning of finite mixture models. *IEEE Transactions on Pattern Analysis and Machine Intelligence* **26**(5), 651-656. (doi:10.1109/TPAMI.2004.1273970).

5. Ruberto T., Polverino G., Porfiri M. 2017 How different is a 3D-printed replica from a conspecific in the eyes of a zebrafish? *Journal of the Experimental Analysis of Behavior*, n/a-n/a. (doi:10.1002/jeab.247).

6. Phamduy P., Polverino G., Fuller R.C., Porfiri M. 2014 Fish and robot dancing together: bluefin killifish females respond differently to the courtship of a robot with varying color morphs. *Bioinspir Biomim* **9**(3), 036021. (doi:10.1088/1748-3182/9/3/036021).

7. Magurran A.E. 2005 *Evolutionary ecology: The Trinidadian guppy* Oxford, Oxford University Press.

**Figures**

**
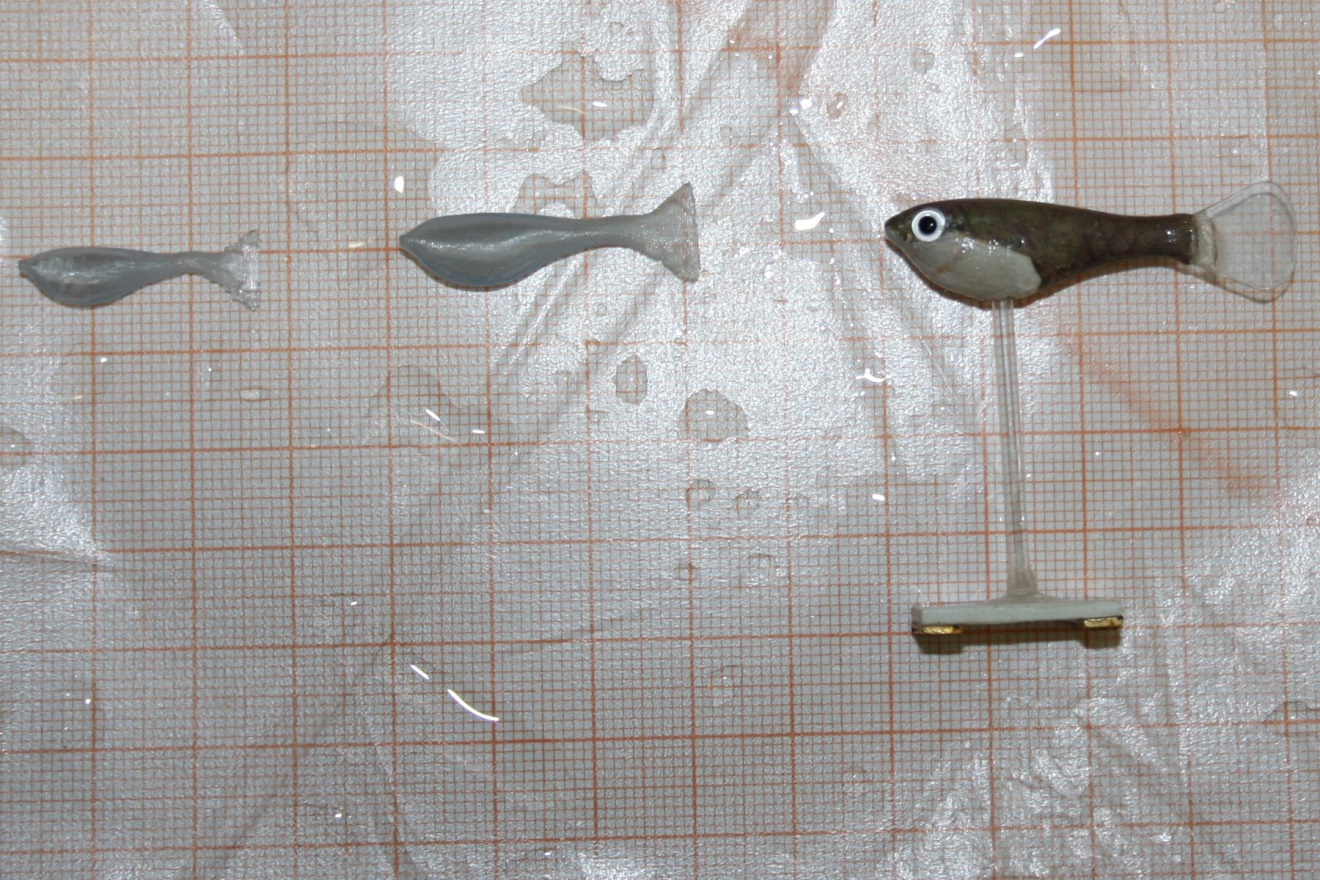
**

**Figure S1:** Photograph of differently-sized replicas. Left (20 mm SL) and middle (25 mm SL) replicas are unprocessed 3D printed blanks that were later on equipped with glass eyes and color-painted as shown for the 30 mm replica on the right.


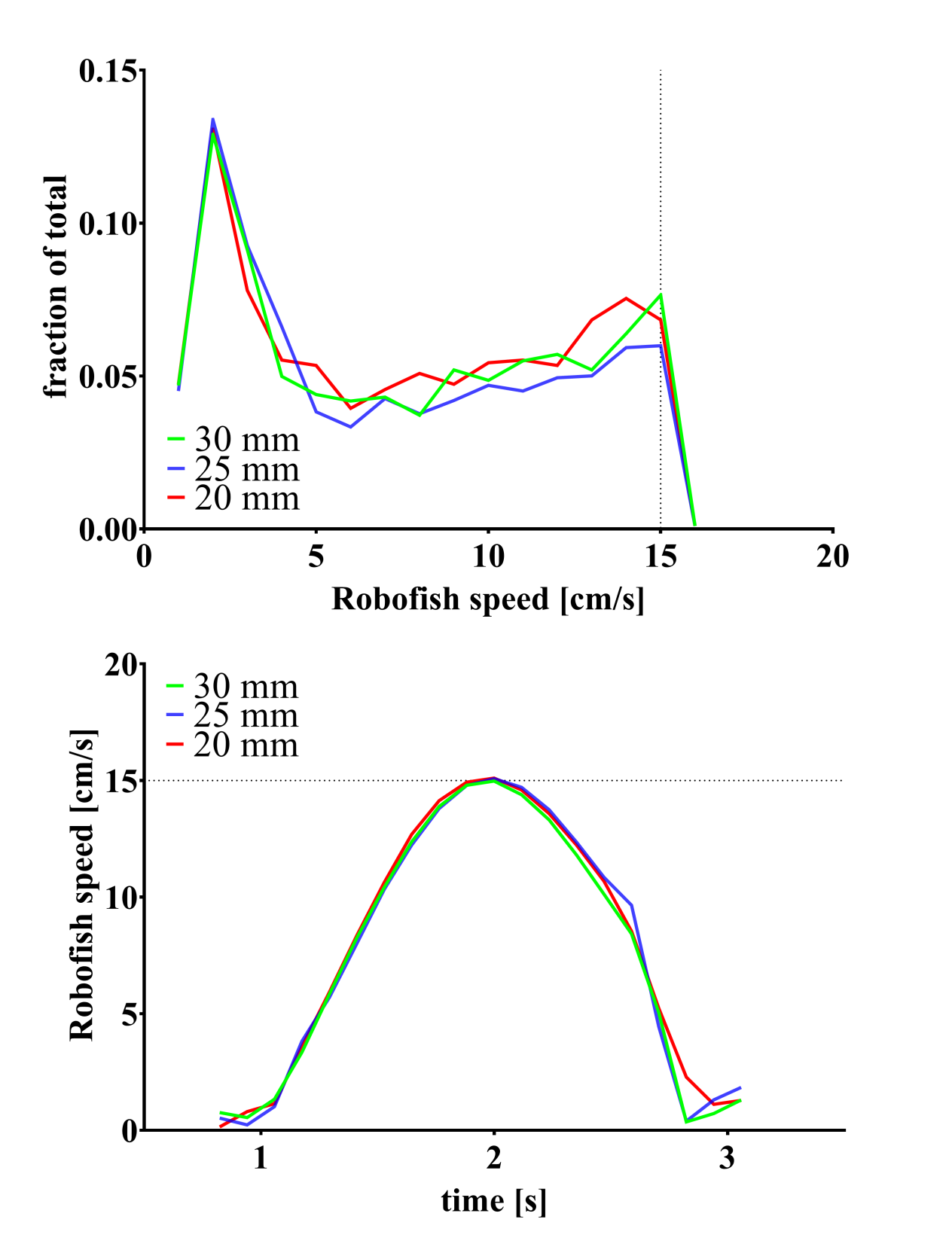


**Figure S2:** Speed distributions during a trial (a) as well as speed profiles (b) of the three differently-sized replicas. In (a) fraction of speeds with a 1 cm/s bin size are shown for a trial run (60 s). There was no significant difference among replicas (Kolmogorov-Smirnoff test: P>0.41). In (b) the speed profiles of Robofish with all three replicas are shown. There was no significant difference in accelerating/decelerating among replicas (Kolmogorov-Smirnoff test: P>0.99). Please note that this pattern of acceleration and deceleration is typically found between two turning points along the zig-zag movement. Maximum speed was set to 15 cm/s (dashed line).
